# Life-Threatening Gastrointestinal Bleeding Revealing a Rare Coexistence of Ampullary Ganglioneuroma and Pancreatic Neuroendocrine Tumor: A Case Report

**DOI:** 10.3390/diagnostics16121778

**Published:** 2026-06-09

**Authors:** Francesk Mulita, Maria Kouroupi, Georgios-Ioannis Verras, Anna-Maria Mitropoulou, Helen Bolanaki, Ioannis Tzimagiorgis, Alexandra Giatromanolaki, Anastasios J. Karayiannakis

**Affiliations:** 1 Second Department of Surgery, Medical School, Democritus University of Thrace, 68100 Alexandroupolis, Greece; med5507@ac.upatras.gr (F.M.); mitropoulou.anma@gmail.com (A.-M.M.); bolanakie@gmail.com (H.B.); tzimagiorgis@hotmail.com (I.T.); 2 Department of Surgery, Medical School, University of Patras, 26504 Patras, Greece; georgiosverras@gmail.com; 3 Department of Pathology, Medical School, Democritus University of Thrace, 68100 Alexandroupolis, Greece; marykouroupi@gmail.com (M.K.); agiatrom@med.duth.gr (A.G.); 4 Department of Surgery, University Hospital Southampton NHS Foundation Trust, Southampton SO16 6YD, UK

**Keywords:** ampullary ganglioneuroma, ampulla of Vater, pancreatic neuroendocrine tumor, gastrointestinal bleeding, whipple procedure

## Abstract

**Background and Clinical Significance:** Ganglioneuromas are rare benign tumors of neural crest origin, with gastrointestinal involvement being uncommon and ampullary localization exceptionally rare. Pancreatic neuroendocrine tumors (NETs) are also uncommon neoplasms with variable biological behavior. The coexistence of these two entities is unusual, particularly in the absence of an identifiable hereditary syndrome. **Case Presentation:** A 38-year-old man presented with hematemesis and multiple episodes of melena over 18 h and was found to have significant anemia with borderline hemodynamic stability. Upper gastrointestinal endoscopy revealed an enlarged, actively bleeding papilla of Vater, and initial hemostasis was achieved with adrenaline injection and endoscopic clipping. However, recurrent massive bleeding developed within 36 h, accompanied by hemodynamic instability. Repeat endoscopy confirmed ongoing hemorrhage, and the patient subsequently underwent emergency pancreaticoduodenectomy. Histopathological examination demonstrated an ampullary ganglioneuroma and an incidental well-differentiated pancreatic neuroendocrine tumor (WHO Grade 2). Surgical margins were negative, and no lymph node metastases were identified. Further evaluation for hereditary endocrine syndromes was unremarkable. The patient remains asymptomatic, with no evidence of recurrence during a 10-year follow-up period. This case highlights the diagnostic and therapeutic challenges associated with rare periampullary tumors. Although ganglioneuromas are typically benign, their anatomical location may result in severe clinical manifestations such as life-threatening bleeding. The coexistence with a pancreatic NET raises questions regarding potential shared pathogenesis, although no genetic syndrome was identified. Limitations of endoscopic management in uncontrolled bleeding and the importance of definitive surgical intervention are emphasized. **Conclusions:** This case highlights an exceptionally rare coexistence of ampullary ganglioneuroma and pancreatic neuroendocrine tumor presenting with life-threatening gastrointestinal bleeding. Although ganglioneuromas are benign, their anatomical location may result in severe clinical manifestations. Early recognition and decisive surgical management are crucial when endoscopic control fails. Favorable long-term outcomes can be achieved following complete resection.

## 1. Introduction

Ganglioneuromas are rare, benign tumors arising from neural crest–derived sympathetic cells and represent the most differentiated end of the neuroblastic tumor spectrum [1,2]. They are typically slow-growing and asymptomatic, most commonly located in the posterior mediastinum, retroperitoneum, or adrenal glands [2,3]. Involvement of the gastrointestinal tract is uncommon, with the colorectum being the most frequently affected site [3,4]. Localization to the ampulla of Vater is notably rare, with only a limited number of cases reported in the literature [4].

Ampullary lesions, irrespective of histology, are of particular clinical significance due to their strategic anatomical location and their potential to cause biliary obstruction, pancreatitis, or gastrointestinal bleeding [5,6]. While adenomas and adenocarcinomas represent the most common ampullary neoplasms, mesenchymal tumors such as ganglioneuromas are rarely encountered in this region [4,5]. When present, they are most often incidental findings; however, symptomatic cases, particularly those presenting with acute upper gastrointestinal hemorrhage, are exceptional [4].

Pancreatic neuroendocrine tumors (NETs) are uncommon neoplasms arising from the diffuse neuroendocrine system of the pancreas, accounting for a small proportion of all pancreatic tumors [7,8]. Their biological behavior ranges from indolent to highly aggressive, and they may present either incidentally or with symptoms related to hormonal hypersecretion or mass effect [7,9]. According to the World Health Organization (WHO) classification, tumor grading based on mitotic count and Ki-67 proliferation index remains a key determinant of prognosis and management [10].

The coexistence of an ampullary ganglioneuroma with a pancreatic neuroendocrine tumor is extraordinarily rare [3,4], as only sporadic cases of ganglioneuromas occurring in association with endocrine-related disorders or hereditary syndromes have been described [11,12,13]. Synchronous presentation with a pancreatic NET in the absence of a defined genetic syndrome remains exceptional [7]. Furthermore, presentation with acute, life-threatening gastrointestinal bleeding adds a layer of clinical complexity and urgency, often necessitating aggressive therapeutic intervention [5].

We report a rare case of ampullary ganglioneuroma coexisting with a well-differentiated pancreatic neuroendocrine tumor, presenting with acute upper gastrointestinal bleeding refractory to endoscopic management and ultimately requiring emergency pancreaticoduodenectomy. This case highlights the diagnostic and therapeutic challenges associated with unusual ampullary lesions. It contributes to the limited body of literature on synchronous neurogenic and neuroendocrine tumors of the periampullary region.

## 2. Case Presentation

A 38-year-old man presented to the emergency department with hematemesis and four episodes of melena over the preceding 18 h. On admission, he was hemodynamically borderline stable, with a blood pressure of 105/60 mmHg and a heart rate of 92 beats per minute. His medical history was unremarkable, with no prior history of peptic ulcer disease or use of non-steroidal anti-inflammatory drugs. Physical examination did not reveal any significant abnormalities.

Initial laboratory investigations revealed marked anemia, with a hemoglobin level of 8.1 g/dL (reference range: 11.0–15.0 g/dL) and a hematocrit of 22.8%. The patient was promptly resuscitated with intravenous crystalloids and colloids, along with transfusion of three units of packed red blood cells.

Following hemodynamic stabilization, an upper gastrointestinal endoscopy was performed. Duodenoscopic evaluation demonstrated an enlarged papilla of Vater with active bleeding (Figure 1). Endoscopic hemostasis was achieved using injection of diluted adrenaline (1:10,000) in combination with application of hemostatic clips (Figure 2A,B).

Subsequent contrast-enhanced abdominal computed tomography revealed a prominent, bulging ampulla of Vater, without evidence of additional intra-abdominal pathology (Figure 3).

Thirty-six hours later, the patient experienced recurrent, massive gastrointestinal bleeding accompanied by hemodynamic instability. After aggressive resuscitation, repeat duodenoscopy confirmed ongoing, profuse bleeding originating from the second part of the duodenum (Figure 4). In view of the failure of endoscopic management and the severity of the hemorrhage, the decision was made to proceed with emergency surgical intervention. Trans-arterial embolization (TAE) was considered as an alternative treatment strategy; however, this option was not available at our institution at the time, as interventional radiology services were not provided as an out of hours service. Patient transfer to a regional referral centre with interventional radiology capability was discussed but was judged to carry an unacceptable risk given the severity of ongoing haemorrhage and the patient’s repeated haemodynamic instability. We acknowledge that in centres where on-site interventional radiology is available, TAE of the gastroduodenal artery would represent a valid and less invasive first-line option before proceeding to surgery.

The patient underwent an emergency pancreaticoduodenectomy (Whipple procedure). Reconstruction was performed with a duct-to-mucosa pancreaticojejunostomy and an antecolic gastrojejunostomy. Pancreaticoduodenectomy was selected as the most appropriate definitive intervention, offering simultaneous haemorrhage control, oncologically complete resection of the ampullary lesion, and accurate histopathological staging, goals that could not reliably be achieved by attempted suture ligation of the bleeding point alone, which would likely have been an insufficient measure altogether given the anatomical location and haemodynamic severity of the haemorrhage.

The postoperative course was uneventful. The surgical drain was removed on postoperative day six, and the patient was discharged in good clinical condition on postoperative day ten.

Histopathological examination identified two distinct lesions. A well-circumscribed lesion measuring 1 cm in diameter was located at the ampulla of Vater. Microscopically, this lesion was composed of spindle-shaped Schwann cells arranged in intersecting fascicles, showing strong immunoreactivity for S100 protein. Scattered mature ganglion cells, positive for neuron-specific enolase (NSE), were also present. There was no evidence of cytological atypia, mitotic activity, or necrosis (Figure 5A–C). There was no invasion past the submucosal plane. These features comply with an ampullary ganglioneuroma.

In addition, an incidental lesion measuring 1.2 cm was identified in the head of the pancreas. Although slightly larger than the ampullary ganglioneuroma, this lesion had not been identified on preoperative contrast-enhanced CT. This is consistent with the well-recognised limited sensitivity of CT for small, non-functional pancreatic NETs, which are frequently iso-attenuating relative to the surrounding parenchyma and may lack a discrete hypervascular blush on arterial-phase imaging, particularly below 2 cm in diameter. Its detection was therefore an incidental intraoperative and histopathological finding. Histological examination revealed a neoplasm composed of uniform, medium-sized round cells with coarse chromatin, inconspicuous nucleoli, and minimal cytological atypia. The tumor exhibited a solid and gyriform architectural pattern within a densely collagenized stroma. Mitotic activity was low, with fewer than 3 mitoses per 10 high-power fields. Immunohistochemical analysis demonstrated diffuse positivity for chromogranin, synaptophysin, and CD56. The Ki-67 proliferation index was less than 10%, consistent with a well-differentiated pancreatic neuroendocrine tumor (WHO Grade 2) (Figure 6A–C).

All surgical margins were free of tumor involvement (R0 resection), and no metastatic disease was identified in the examined peripancreatic lymph nodes.

Given the known association of ganglioneuromas with hereditary syndromes, further evaluation for multiple endocrine neoplasia type 2 and PTEN hamartoma tumor syndrome (Cowden’s disease) was undertaken. Neck ultrasonography showed no abnormalities of the thyroid or parathyroid glands, thyroid function tests were within normal limits, and functional imaging with ^111^In-octreotide scintigraphy did not demonstrate any pathological uptake. No other features of neurofibromatosis syndromes were present, and the patient was referred to the Clinical Geneticist for germline testing, which did not result in a genetic syndrome diagnosis. Therefore, this was deemed a sporadic case of rare tumors of neural crest origin.

At long-term follow-up, the patient remains asymptomatic, with no clinical or radiological evidence of recurrence over 10 years. The latest follow-up CT scan performed at 10 years was unremarkable.

## 3. Discussion

A review of the recent literature (last 10 years) shows that ampullary ganglioneuromas and related entities, such as gangliocytic paragangliomas, are rare tumors, most commonly located in the periampullary region. Fewer than 60 cases have been reported in the literature in this period, with gastrointestinal bleeding being the most frequent presenting symptom. However, life-threatening hemorrhage requiring emergency surgery remains exceptionally rare.

A summary of representative cases from the recent literature is presented in Table 1. Given the rarity of strictly ampullary lesions, selected periampullary cases were included for comparison.

A review of the available literature suggests that periampullary gangliocytic paragangliomas most commonly present in middle-aged patients, with a slight male predominance. Tumor size is typically less than 3 cm, and most lesions are confined to the submucosa at diagnosis. Endoscopic management has been increasingly reported in recent years; however, surgical resection remains the standard approach in cases of diagnostic uncertainty, suspected malignancy, or severe complications such as uncontrolled bleeding [1,3,18,19,20,21,22,23,24].

Ganglioneuromas, composite gangliocytomas and neuroendocrine tumor (CoGNET) are rare tumors arising from neural crest–derived elements, most frequently located in the second portion of the duodenum and particularly in the periampullary region [20,21,22]. Although traditionally considered benign, their biological behavior appears more heterogeneous than previously thought, as cases with lymph node involvement and, rarely, distant metastases have been described [20,23,24]. Histologically, these tumors may demonstrate a characteristic triphasic architecture consisting of epithelioid neuroendocrine cells, spindle Schwann-like cells, and ganglion cells, which contributes to diagnostic challenges and ongoing debate regarding their histogenesis [20,21,22].

From a clinical perspective, most lesions are asymptomatic or incidentally detected. However, gastrointestinal bleeding represents the most frequent presenting symptom, reported in a substantial proportion of cases [23,24]. In the present case, the patient developed an acute, life-threatening hemorrhage requiring urgent intervention. Notably, the severity of bleeding appeared disproportionate to the relatively small size of the ampullary lesion, emphasizing the clinical impact of tumor location in this region. Similar presentations, although uncommon, have been reported [14,15]. Other manifestations, including abdominal pain, anemia, biliary obstruction, and pancreatitis, further expand the clinical spectrum [23,24].

Compared to previously reported cases, our patient presented with unusually severe and rapidly recurrent hemorrhage despite initial endoscopic hemostasis. In most published cases, bleeding is either self-limited or successfully managed endoscopically, whereas the need for emergency pancreaticoduodenectomy is rarely described [14,17,18,19].

The diagnostic evaluation of periampullary ganglioneuromas remains challenging. Imaging modalities such as computed tomography and endoscopic ultrasound are useful for lesion localization but lack specificity. In addition, endoscopic biopsies are often inconclusive due to the submucosal nature of these tumors, and misclassification as neuroendocrine or other subepithelial lesions is not uncommon [23,24]. In our case, a definitive diagnosis was only established following surgical resection, highlighting the limitations of preoperative assessment in such settings.

Endoscopic ultrasound (EUS) may provide additional information regarding the layer of origin and vascularity of the lesion; however, its diagnostic accuracy remains limited in distinguishing ganglioneuromas from other subepithelial tumors. Fine-needle aspiration is often inconclusive due to the heterogeneous cellular composition of these tumors, further complicating preoperative diagnosis [25,26,27,28].

A particularly noteworthy aspect of our case is the synchronous presence of an ampullary ganglioneuroma and a pancreatic neuroendocrine tumor in the absence of a hereditary syndrome. To our knowledge, such synchronous occurrences have been described in only an extremely limited number of cases. The pancreatic NET most likely represents an incidental finding, given the absence of any identifiable hereditary syndrome and the lack of a defined mechanistic link between these two entities. We therefore use the term “coexistence” in a purely descriptive and clinicopathological sense, to denote the simultaneous presence of two rare tumours in the same patient, without implying a causal or syndromic relationship. Any allusion to a shared pathogenetic pathway should be understood as speculative and hypothesis-generating rather than as an established biological association, particularly given the high prevalence of small, incidental NETs in the general population.

This raises the possibility of a shared differentiation pathway between neurogenic and neuroendocrine elements, as suggested by recent studies on composite gangliocytic tumors. Recent molecular and histopathological studies suggest that these tumors may arise from pluripotent neural crest–derived cells capable of divergent differentiation. This may explain the coexistence of neurogenic and neuroendocrine components in the same anatomical region. Recent studies on composite gangliocytoma/neuroma and neuroendocrine tumors (CoGNET) have demonstrated overlapping morphological, immunohistochemical, and molecular features, supporting this concept [20,21,22]. Earlier reports describing mixed endocrine–neurogenic tumors in the ampullary region further reinforce this hypothesis [29]. Nevertheless, in the absence of a defined hereditary syndrome, it remains difficult to determine whether this association is coincidental or reflects an underlying biological link [20,22].

Despite their generally favorable prognosis, these tumors cannot be regarded as entirely benign. Large clinicopathological series have reported lymph node metastasis in approximately 6–12% of cases, particularly in tumors demonstrating increased size, deeper invasion, or lymphovascular involvement [20,23,24]. Rare cases of distant metastasis and even fatal outcomes have also been documented [19,29]. These observations have led some authors to classify these lesions as tumors of uncertain or low-grade malignant potential. Consequently, complete resection and careful long-term follow-up are generally recommended [23,29].

Management should be tailored to tumor characteristics and clinical presentation. Endoscopic resection may be appropriate for small, localized lesions confined to the submucosa [24]. However, this approach is limited by the potential for incomplete excision and the inability to assess lymph node status. In our patient, initial endoscopic hemostasis was unsuccessful, and ongoing hemorrhage necessitated emergency surgical intervention. Pancreaticoduodenectomy allowed definitive control of bleeding, complete tumor removal, and accurate histopathological evaluation. Recently, minimally invasive endoscopic techniques have expanded the therapeutic options for selected periampullary lesions. Underwater endoscopic mucosal resection (UEMR) has been reported as a safe and effective approach for en bloc resection of small periampullary subepithelial tumors, although experience remains limited and careful patient selection is required [30].

The long-term outcome in our case is also informative. The patient remains asymptomatic with no evidence of recurrence after 10 years of follow-up. This finding is consistent with previously reported favorable outcomes following complete resection, even in cases with complex histological features or synchronous lesions [19,20,24]. It also supports the role of surgical management as a definitive treatment strategy in selected high-risk or emergent scenarios.

Importantly, upon confirmation of the lesion’s histology, the importance of further genetic workup of the patient with next-generation sequencing, for genetic syndromes that can be associated with such neoplasms, including MEN1, RET pathway mutations, or PTEN mutations that also predispose to a high incidence of several malignancies, for which the patients should be screened. Although ganglioneuromas are generally considered benign, their location in the ampullary region may lead to severe and rapidly evolving clinical manifestations [23,24]. The synchronous presence of a pancreatic neuroendocrine tumor raises the possibility of overlapping neurogenic and neuroendocrine differentiation, as suggested by recent studies on composite neoplasms. However, in the absence of an identifiable hereditary syndrome, the exact relationship between these entities remains uncertain [20,21,22].

A review of the available literature suggests that while most periampullary ganglioneuromas follow an indolent course, a subset may present with severe complications such as massive gastrointestinal bleeding. Our case further expands this spectrum by demonstrating that even small lesions may result in hemodynamically significant hemorrhage requiring emergency surgical management [31,32,33].

Compared to previously reported cases, our case is distinguished by the severity and rapid recurrence of hemorrhage, necessitating emergency pancreaticoduodenectomy despite initial endoscopic control. This highlights a rare but important clinical scenario and expands the current understanding of the potential behavior of periampullary ganglioneuromas.

This report has certain limitations. As a single case report, it does not allow firm conclusions regarding the relationship between ampullary ganglioneuromas and pancreatic neuroendocrine tumors. In addition, molecular or genetic analyses were not performed, which might have provided further insight into potential shared pathogenetic mechanisms. Nonetheless, the rarity of this presentation, combined with the detailed clinical and pathological documentation, adds useful information to the existing literature.

Future studies focusing on molecular profiling and larger case series are required to better define the biological behavior and optimal management strategies of these rare tumors. Improved understanding of their pathogenesis may also help clarify the relationship between neurogenic and neuroendocrine components, particularly in cases presenting with synchronous lesions [34,35].

## 4. Conclusions

Our case describes a rare coexistence of ampullary ganglioneuroma and pancreatic neuroendocrine tumor presenting with life-threatening gastrointestinal bleeding. Although ganglioneuromas are generally considered benign, their anatomical location in the ampullary region may lead to severe and rapidly evolving clinical manifestations. It also illustrates the limitations of endoscopic management in the setting of uncontrolled bleeding. In addition, this case highlights the need for timely surgical intervention when initial measures fail or when diagnostic uncertainty persists. The favorable long-term outcome observed in this patient suggests that complete surgical resection can be curative, even in complex and emergent scenarios. It adds to the limited literature on rare periampullary tumors and supports the importance of individualized management and careful follow-up. Further studies are needed to define the biological relationship between these entities better.

## Figures and Tables

**Figure 1:**
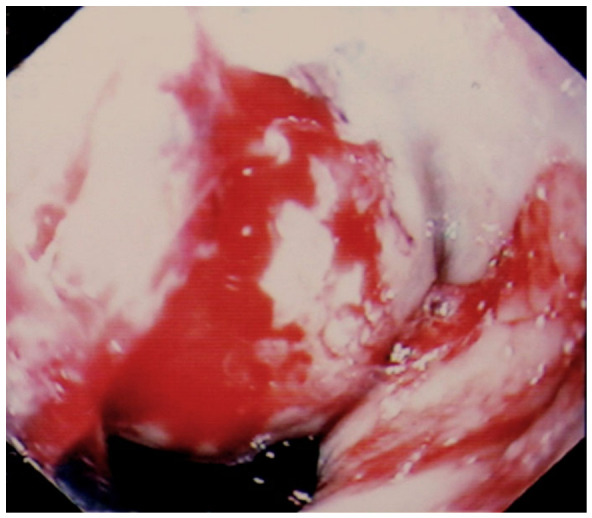
Endoscopic appearance of the ampulla of Vater. Enlarged and edematous papilla with active arterial bleeding at presentation. The lesion appears congested with overlying mucosal disruption.

**Figure 2:**
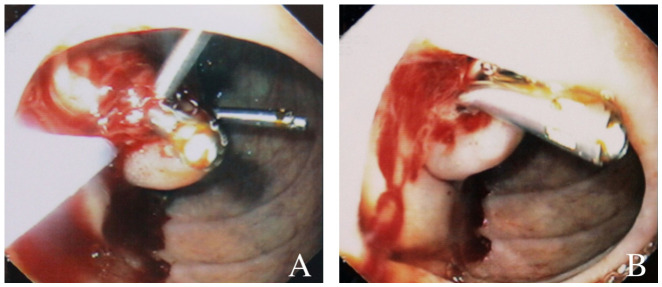
Endoscopic hemostatic intervention. (**A**) Active bleeding from the ampulla of Vater during endoscopic intervention, with visualization of the bleeding point. (**B**) Successful application of endoscopic clips at the ampullary region, achieving temporary control of hemorrhage.

**Figure 3:**
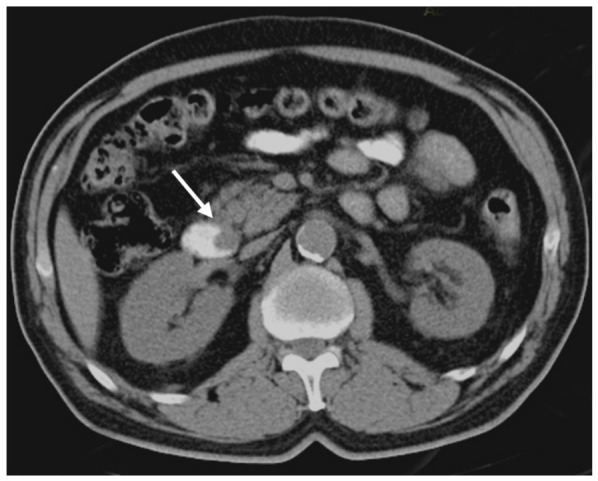
Contrast-enhanced computed tomography of the abdomen. Axial view demonstrating a prominent, bulging ampulla of Vater (arrow) without direct evidence of pancreatic mass or distant pathology. This was later confirmed to be caused by the CT-occult ganglioneuroma lesion.

**Figure 4:**
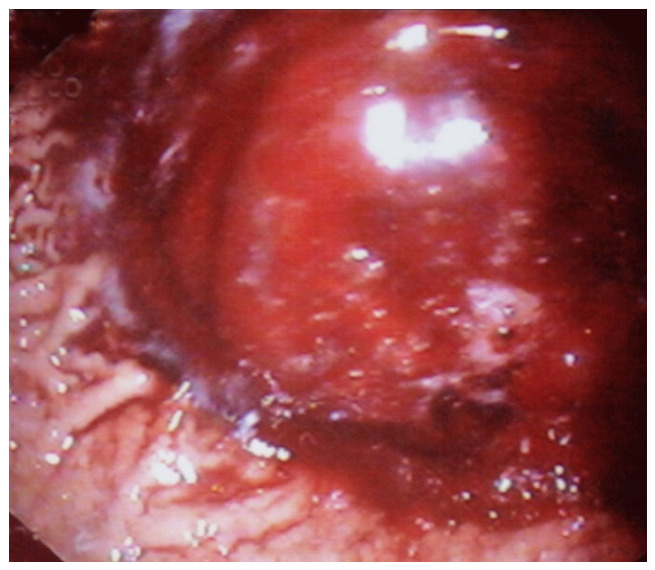
Recurrent gastrointestinal bleeding. Repeat duodenoscopy showing profuse, active bleeding originating from the second portion of the duodenum. Visualization is limited due to ongoing hemorrhage despite prior endoscopic treatment.

**Figure 5:**
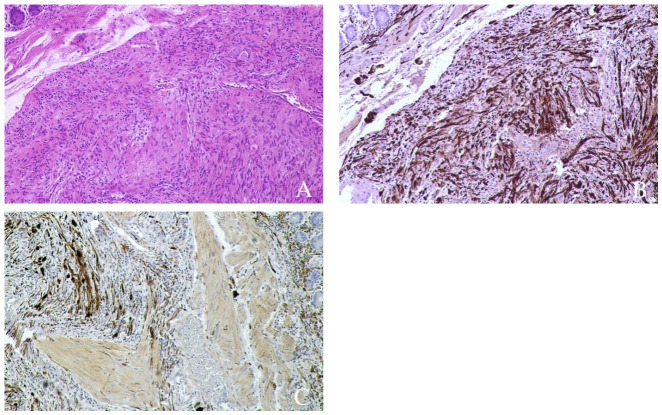
Histopathological features of ampullary ganglioneuroma. (**A**) Hematoxylin and eosin staining showing a submucosal, well-organized proliferation of spindle cells arranged in fascicles, without cytological atypia (×10). (**B**) Immunohistochemical staining for S100 demonstrating strong and diffuse positivity in Schwann cells, confirming neural crest origin (×10). (**C**) Neuron-specific enolase (NSE) immunostaining highlighting scattered mature ganglion cells within the lesion (×10).

**Figure 6:**
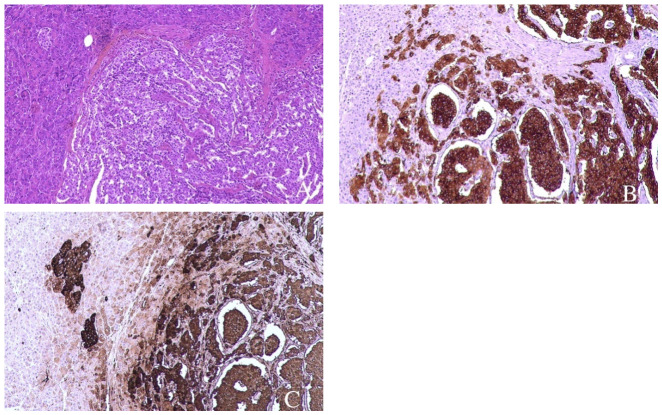
Histopathological and immunohistochemical features of the pancreatic neuroendocrine tumor. (**A**) Hematoxylin and eosin staining demonstrating a well-circumscribed neoplasm composed of uniform cells with round nuclei and finely granular (‘salt-and-pepper’) chromatin arranged in solid and trabecular patterns within a delicate fibrovascular stroma (original magnification ×10). (**B**) Immunohistochemical staining for synaptophysin showing diffuse cytoplasmic positivity, supporting neuroendocrine differentiation (×10). (**C**) Chromogranin A immunostaining demonstrating strong and diffuse positivity in tumor cells (×10).

**Table 1:** Summary of representative reported cases of ampullary and periampullary gangliocytic paraganglioma (last 10 years).

Author, Year	Age, Sex	Location	Presentation	Treatment	Outcomes
Adams et al., 2018 [14]	49, Female	Duodenum (periampullary)	Upper GI bleeding	Pancreaticoduodenectomy	Good outcome
Arif et al., 2020 [15]	47, Male	Periampullary duodenum	Melena, anemia	Surgical local excision	Uneventful, no recurrence
Choi et al., 2022 [16]	46, Female	Ampulla of Vater	Anemia	Pancreaticoduodenectomy + LN dissection	LN metastasis present, good outcome
Cai et al., 2023 [17]	54, Female	Ampulla of Vater	Melena	Endoscopic papillectomy + ERCP	Good outcome
Takada et al., 2025 [18]	Case series (6 patients)	Ampulla of Vater	Variable (bleeding/incidental)	Endoscopic papillectomy	No recurrence during follow-up

## Data Availability

The original contributions presented in this study are included in the article. Further inquiries can be directed to the corresponding author.
